# Real-World Evaluation of Letermovir Use in Kidney Transplant Recipients: Drug Interactions, Safety, and Impact on Renal Function

**DOI:** 10.3389/ti.2025.15371

**Published:** 2025-12-04

**Authors:** Ilies Benotmane, Betoul Schvartz, Cyril Garrouste, Florence Runyo, Charlotte Boud’hors, Gauthier Flahaut, Clément Danthu, Dominique Bertrand, Sophie Caillard

**Affiliations:** 1 Department of Nephrology, Dialysis and Transplantation, Strasbourg University Hospitals, Strasbourg, France; 2 Laboratoire d’ImmunoRhumatologie Moléculaire, INSERM UMR_S1109, Fédération de Médecine Translationnelle de Strasbourg (FMTS), Université de Strasbourg, Strasbourg, France; 3 Department of Nephrology and Transplantation, University of Reims, Reims, France; 4 Department of Nephrology and Transplantation, University of Clermont Ferrand, Clermont Ferrand, France; 5 Service de Néphrologie et Transplantation Adultes, Hôpital Universitaire Necker, APHP, Paris, France; 6 Department of Nephrology and Transplantation, University of Angers, Angers, France; 7 Service de Néphrologie, Dialyse et Transplantation, CHU Amiens-Picardie, Amiens, France; 8 Department of Nephrology and Transplantation, University of Limoges, Limoges, France; 9 Department of Nephrology and Transplantation, University of Rouen, Rouen, France

**Keywords:** cytomegalovirus, letermovir, drug interactions, kidney transplant, kidney function

Dear Editors,

Letermovir is approved for primary prophylaxis of cytomegalovirus (CMV) infection in seropositive donor/seronegative kidney transplant recipients (KTRs) [1, 2]. Letermovir inhibits CYP3A4, raising the risk of interactions with calcineurin inhibitors (CNIs) and mTOR inhibitors (mTORi) [3]. Real-world data regarding these interactions are limited [4]. This retrospective multicenter study evaluated letermovir-immunosuppressant interactions and assessed letermovir safety and efficacy.

Twenty-six KTRs were included. Detailed methods are provided in the Supplementary Material, and patient characteristics in Supplementary Table S1. Letermovir was initiated at a median of 135 days [IQR: 109–139] post-transplantation for primary prophylaxis (patients with a history of or current valganciclovir resistance or intolerance, n = 5), 264 days [192–397] for curative treatment (n = 11), and 296 days [252–423] for secondary prophylaxis (n = 10). At data cutoff, five patients remained on letermovir; one died of CMV disease and another lost graft function. Among the rest, median treatment duration was 151 days [66–361].

In the 16 patients receiving tacrolimus, the median daily dose significantly decreased from 3.6 mg [2.6–7.1] before letermovir to 2.3 mg [1–4.8] during treatment (p = 0.002, Figure 1A; Supplementary Table S2), corresponding to a median 33% dose reduction (range: 0%–75%). Similar findings have been reported in transplant recipients, with most studies recommending a 30%–50% dose reduction [5–7]. Two patients also receiving CYP3A4 inhibitors (lansoprazole, amiodarone) had among the largest tacrolimus dose reductions—74% and 60%—suggesting a cumulative effect. No association was found between dose reduction and body mass index (Spearman ρ = −0.04, p = 0.87), or with tacrolimus formulation (immediate-release: 33% reduction; melt-dose: 38%; prolonged-release: 0%; p = 0.41). Tacrolimus trough levels significantly increased from a median of 6.2 ng/mL [5.1–9.3] to 8.3 ng/mL [7.3–13.3] during letermovir treatment (p = 0.006, Figure 1B).

**FIGURE 1:**
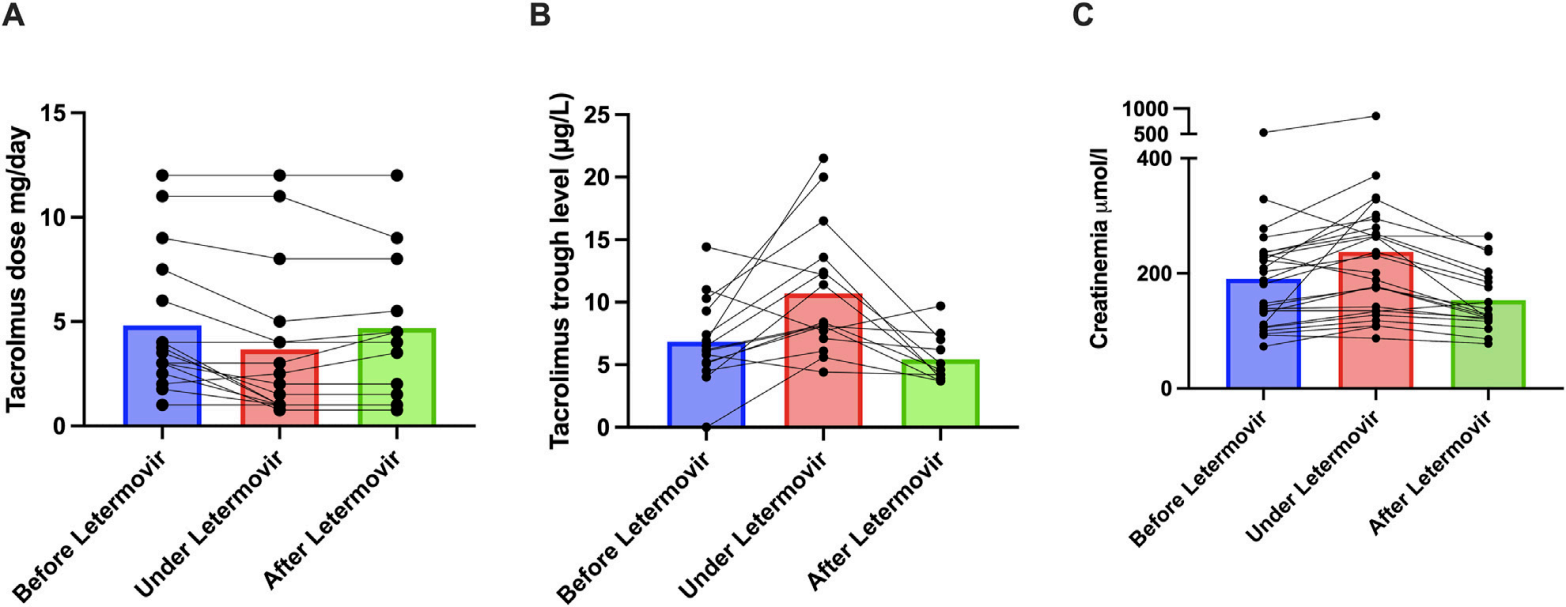
Impact of Letermovir on Tacrolimus dosage, Tacrolimus residual levels and renal function. **(A)** Changes in tacrolimus dosage before (n = 16), during (n = 16), and after stopping letermovir (n = 12). **(B)** Changes in tacrolimus residual levels before (n = 15), during (n = 16), and after stopping letermovir (n = 11). The tacrolimus trough level was below the limit of detection for one patient therefore, a value of zero was assigned. **(C)** Creatinine in µmol/L before (n = 26), during (n = 26), and after (n = 19) treatment with letermovir. Each dots represents a patient. The plot represents the median (bar).

Among 12 patients with post-letermovir treatment data, tacrolimus daily doses remained stable (3.5 mg [1.6–7.3] vs. 4.3 mg [1.6–7.4], p = 0.88), but trough levels significantly decreased after discontinuation (from 8.1 ng/mL [7.1–12.4] to 7 ng/mL [3.9–7.0], p = 0.01).

In the 5 patients receiving ciclosporin, the median dose decreased from 200 mg/day [125–200] to 100 mg/day [90–200], without reaching statistical significance (p = 0.25). In four patients with available through levels, concentrations increased from 71 ng/mL [55–126] to 169 ng/mL [152–406] (p = 0.13). In three patients with post-letermovir treatment data, doses remained unchanged in two and doubled in one. Notably, previous pharmacokinetic data showed a 1.7-fold increase in ciclosporin AUC with letermovir [3].

Among four patients on everolimus, one discontinued the drug shortly after starting letermovir. In the remaining three, trough levels increased (11.3, 10.8, and 9.1 ng/mL), prompting 50% dose reductions in two cases. In healthy volunteers, letermovir increased everolimus AUC by 3.4-fold [3].

An unanticipated observation was a transient 18% increase in serum creatinine following letermovir initiation from 185 μmol/L [110–235] to 216 μmol/L [135–284] (p = 0.0006), with 17 of 26 patients (65%) meeting KDIGO 1 criteria for acute kidney injury (AKI, defined as a ≥26 μmol/L increase). Seven of them also experienced gastrointestinal side effects that may have led to functional AKI. In 19 patients with post-letermovir treatment data, creatinine increased from 143 μmol/L [107–230] to 178 μmol/L [128–264] during treatment (p = 0.03), and then decreased to 126 μmol/L [120–193] after discontinuation (p = 0.0002), indicating reversibility (Figure 1C). No correlation was found between creatinine increase and tacrolimus peak levels (Spearman ρ = −0.08, p = 0.72), and creatinine elevation occurred also in all five patients not on CNIs (ranging from 28 μmol/L to 123 μmol/L). Possible mechanisms include inhibition of renal tubular OAT3 transporters by letermovir impairing creatinine elimination [8] or gastrointestinal symptoms leading to functional AKI. In the trial by Limaye et al. [9], AKI occurred in only 6.8% of patients receiving letermovir, similar to the valganciclovir group. As letermovir was initiated early post-transplant—when renal function is recovering—minor creatinine increases may have been difficult to detect.

Gastrointestinal adverse events, including diarrhea and vomiting, were reported in 9 patients (35%), consistent with earlier reports [9, 10]. These events were not associated with CNI exposure (7/9 vs. 14/17, p > 0.99) or tacrolimus trough levels (8.3 vs. 9.8 ng/mL, p = 0.7).

Letermovir was used for prophylaxis in 15 patients—10 due to valganciclovir-induced cytopenia and five for a prior history of valganciclovir resistance or poor virologic response. CMV replication occurred in three patients on secondary prophylaxis. Resistance was excluded in one case; two were not tested. These findings support cautious off-label use of letermovir for secondary prophylaxis in select cases [10].

Letermovir was used as curative therapy in 11 patients, mainly for CMV resistant to first-line antivirals (n = 10), and often in combination with other anti-CMV agents (n = 6). Treatment was initiated in a context of low viral load (median 3.49 log_10_ IU/mL [3.22–3.72]). Two patients experienced viral load increases (from 3.3 to 5.6 log_10_ IU/mL and from 3.2 to 4.3 log_10_ IU/mL, respectively), and both developed confirmed letermovir resistance. One of these patients died from CMV disease.

Because letermovir is unapproved for curative therapy and carries a low genetic barrier to resistance, it should only be considered in selected low viral load refractory cases, as a last-resort option in combination with other antiviral agents.

Despite limitations—including retrospective design, small sample size, and lack of standardized therapeutic drug protocols—this study suggests that letermovir use is associated with significant pharmacokinetic interactions with CNIs and mTORi, warranting close drug level monitoring during initiation and discontinuation. Clinicians should also be alert to the potential for renal function decline hopefully reversible.

## Data Availability

The raw data supporting the conclusions of this article will be made available by the authors, without undue reservation.
