# Supplementary material for: Real-World Evaluation of Letermovir Use in Kidney Transplant Recipients: Drug Interactions, Safety, and Impact on Renal Function
Source: Transpl Int. 2025 Dec 4;38:15371. doi: 10.3389/ti.2025.15371 (PMC12711588; doi:10.3389/ti.2025.15371)

**Supplementary Material**

A retrospective, multicenter observational study was conducted across eight French kidney transplant centers. All adult kidney transplant recipients (KTRs) who received letermovir between August 2020 and June 2024 were included. Patients had provided informed consent for inclusion in the ASTRE database, which collects clinical and biological data from these centers (DR-2012-518).

Baseline demographic, clinical, and transplantation-related characteristics were recorded, including donor and recipient cytomegalovirus (CMV) serostatus at the time of transplantation, CMV management strategies, and any documented episodes of CMV replication. Six of the eight centers employed universal prophylaxis. One center restricted prophylaxis to donor-positive/recipient-negative (D+/R−) patients and to R+ recipients treated with T-cell depleting agents. Another center used universal prophylaxis for both D+/R− patients and R+ recipients receiving T-cell depleting agents, while applying a preemptive strategy guided by CMV ELISPOT results in R+ patients treated with basiliximab.

Letermovir was used off-label in this cohort. Its indication was categorized as primary prophylaxis, secondary prophylaxis, or curative treatment. Additional data were collected on the time from transplantation to letermovir initiation, dosing regimens, and treatment duration.

Immunosuppressive therapy and renal function were comprehensively documented, including the specific agents used, dosing protocols, and trough levels of calcineurin inhibitors (CNIs) and mammalian target of rapamycin (mTOR) inhibitors before, during, and after letermovir therapy. Pre-treatment data were defined as follows: for clinical variables such as immunosuppressant dosing, values at letermovir initiation were used; for laboratory parameters, the closest available pre-treatment value was selected, with a median of 0.5 days (IQR 0–6.25) prior to letermovir initiation.

During letermovir therapy, all available data on drug dosages, trough levels, and renal function were reviewed. The nadir of immunosuppressive drug dosages was observed after a median of 15 days (IQR 7.8–46) following letermovir initiation, while peak trough levels occurred after a median of 13 days (IQR 7–42). Peak serum creatinine levels were noted after a median of 49.5 days (IQR 13.3–82.3). Post-treatment data were collected for a median duration of 27 days (IQR 21–44) for trough level and a median duration of 30 days (IQR 18–44) for creatinine. Immunosuppressive regimens were adjusted at the discretion of individual clinicians when clinically indicated, particularly in the absence of standardized recommendations. Data on concomitant medications with potential interactions (e.g., statins, antiarrhythmics, anticoagulants, and antidiabetic agents) were also recorded. Adverse events potentially attributable to letermovir or drug-drug interactions were extracted from medical records.

Letermovir efficacy was evaluated based on its ability to prevent CMV replication or disease during prophylaxis, as well as its performance in curative settings. In curative use, efficacy assessment included viral load kinetics, time to viral clearance, and clinical outcomes. Treatment failure was defined as CMV infection refractory to therapy, characterized by a ≤1 log₁₀ reduction in plasma or whole blood viral load after ≥2 weeks of treatment, or by the onset or progression of CMV disease.^1^

Continuous variables were expressed as medians with interquartile ranges, and categorical variables as counts and percentages. The Wilcoxon matched-pairs signed-rank test was used for comparisons between paired observations, and the Kruskal–Wallis test for unpaired comparisons. Associations between continuous variables were assessed using Spearman’s rank correlation.

**Reference**

1. Kotton CN, Kumar D, Manuel O, et al. The Fourth International Consensus Guidelines on the Management of Cytomegalovirus in Solid Organ Transplantation. *Transplantation*. 2025;109(7):1066-1110.

| **Table S1 : patients characteristics** |
| --- |

|  | **Overall** | **Primary prophylaxis** | **Secondary prophylaxis** | | **Curative** |
| --- | --- | --- | --- | --- | --- |
|  | N = 26 | N = 5 | N = 10 | | N = 11 |
| Male | 16 (62%) | 3 (60%) | 5 (50%) | | 8 (73%) |
| Age | 53 (44, 64) | 47 (46, 54) | 52 (42, 68) | | 58 (44, 70) |
| First transplantation | 18 (69%) | 3 (60%) | 7 (70%) | | 8 (73%) |
| Induction* |  |  |  | |  |
| ATG | 13 (52%) | 2 (40%) | 5 (56%) | | 6 (55%) |
| Simulect | 12 (48%) | 3 (60%) | 4 (44%) | | 5 (45%) |
| Calcineurin inhibitor |  |  |  | |  |
| Tacrolimus | 16 (62%) | 3 (60%) | 7 (70%) | | 6 (55%) |
| Ciclosporin | 5 (19%) | 1 (20%) | 2 (20%) | | 2 (18%) |
| Tacrolimus formulation |  |  |  | |  |
| Immediate relase | 3 (19%) | 0 (0%) | 1 (14%) | | 2 (33%) |
| meltodose | 6 (38%) | 2 (67%) | 2 (29%) | | 2 (33%) |
| Prolonged release | 7 (44%) | 1 (33%) | 4 (57%) | | 2 (33%) |
| Mycophenolate Mofetil | 15 (58%) | 5 (100%) | 5 (50%) | | 5 (45%) |
| Everolimus | 4 (15%) | 0 (0%) | 1 (10%) | | 3 (27%) |
| Belatacept | 3 (12%) | 1 (20%) | 1 (10%) | | 1 (9.1%) |
| Steroids | 23 (88%) | 4 (80%) | 9 (90%) | | 10 (91%) |
| CMV Serological status* |  |  |  | |  |
| D+/R- | 17 (68%) | 4 (80%) | 6 (67%) | | 7 (64%) |
| D+/R+ | 4 (16%) | 0 (0%) | 2 (22%) | | 2 (18%) |
| D-/R+ | 2 (8.0%) | 1 (20%) | 1 (11%) | | 0 (0%) |
| D-/R- | 2 (8.0%) | 0 (0%) | 0 (0%) | | 2 (18%) |
| CMV primary prophylaxis | 23 (88%) | 5 (100%) | 10 (100%) | | 8 (73%) |
| CMV primary prophylaxis initiation delays in days | 5 (0, 9) | 6 (2, 7) | 3 (0, 5) | | 9 (5, 164) |
| Letermovir dose (mg)** |  |  |  | |  |
| 240 | 9 (35%) | 1 (20%) | 4 (40%) | | 4 (36%) |
| 480 | 17 (65%) | 4 (80%) | 6 (60%) | | 7 (64%) |
| Creatinine at letermovir initiation, µmol/l | 185 (111, 234) | 223 (106, 278) | 146 (135, 203) | | 209 (111, 236) |
| CNI: calcineurin inhibitors, CMV : cytomegalovirus | | | |  |  |
| * missing data for one patient  **Four patients received a lower-than-recommended dose of letermovir (i.e., 240 mg instead of 480 mg) while being treated with tacrolimus | | | |  |  |

**Table S2 : Patient-Specific Immunosuppressant Dosing, Trough Concentrations, and Timing in Relation to Letermovir Initiation and Termination**

mTORi : mTOR inhibitor


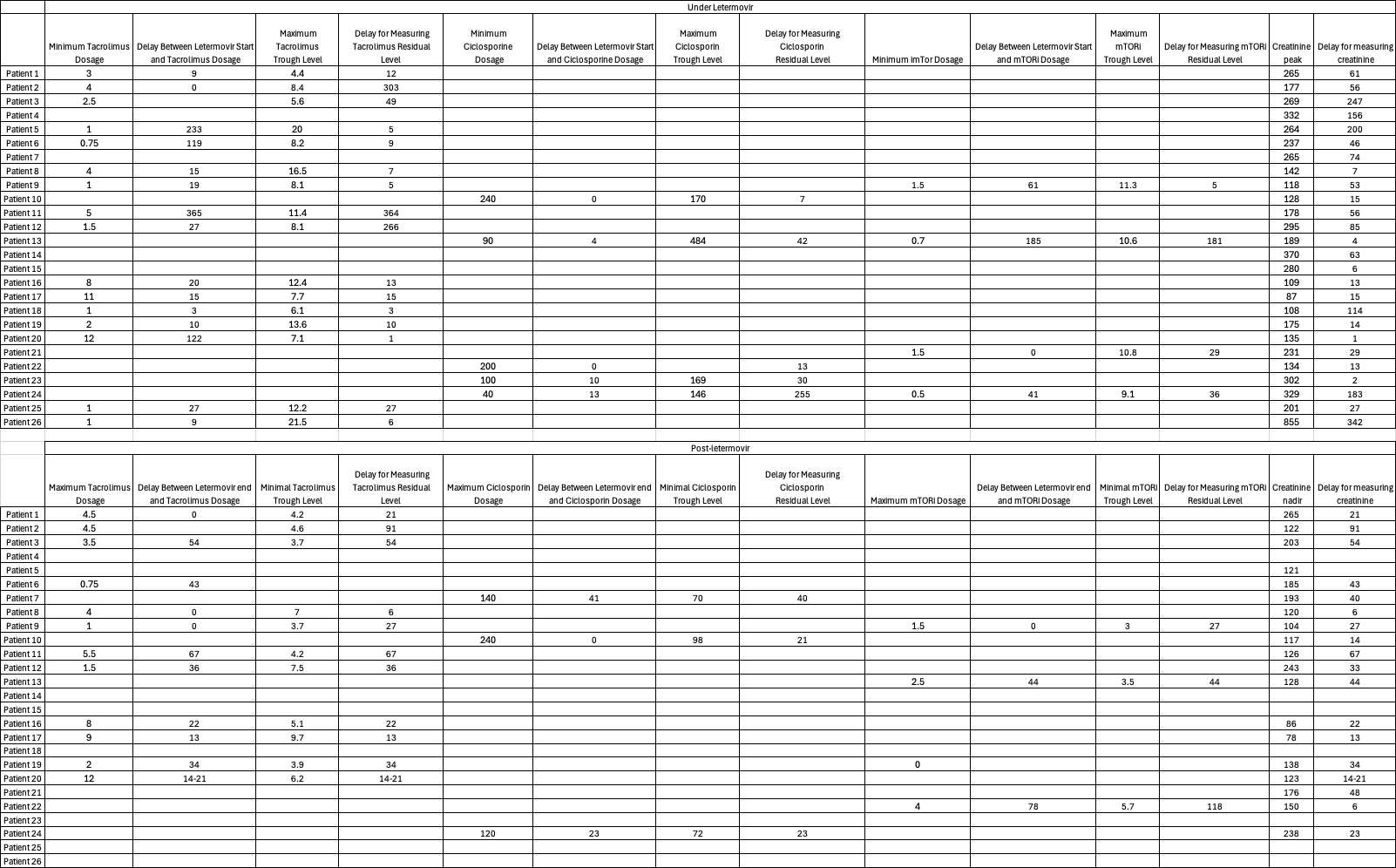

Supplement: Supplementary file 1 [file DataSheet1.docx]
